# Varieties

**Published:** 1858-06

**Authors:** 


### VARIETIES.

A DYSPEPTIC Bachelor, in reply to our advice, that if he must eat pies, the under crust should be baked first, and the prepared fruit spread over it, writes: "How many women do you suppose would make pies as you suggest? They think they know, and when they will they will, and when they won't they won't, and that's the end of it. I shall, therefore, not eat any pies at present. I am glad you tell me the nature of my troubles so clearly. I never knew what Dyspepsia was, until I read it a few months ago in the JOURNAL OF HEALTH; and ever since, I have had a growing conviction that I have been overtasking my stomach." We may safely say, that this gentleman is one of a multitude, who are doing the same thing.

THE incumbent of a literary institution writes: "Your Journal is a welcome visitor to my family. My wife reads it through, quotes it daily, and urges all the house to read and practice its precepts. I read parts of it in my school, and make out of it text, testimony, and commentary. You are doing a good work. May you long live and continue your philanthropic labors. You write so that people love to read what you say; they understand and remember it."

A WALL-STREETER objects to our advertisements, and writes: "I have no idea of paying my money for binding up other people's advertisements." Being a bachelor, he has no idea of baby conveniences, has no taste for the luxurious carpetings of Mr. LANDON, nor for the far-famed sweet-toned pianos of WORCESTER; PRINCE'S melodeons carry no music into his soul; the brightly-burning "Cannel" of TRUSLOW BROTHERS fails to warm up his frozen heart. What a dreadful thing it must be to be a crusty, grumpy old bach, to look at Beauty's eyes through yellow spectacles, to turn away with fretfulness at the prattle of children, to live in a region where there is no song, no smile, no flower—a clime so bleak, and desolate, and drear, and dark, that if the dull eye can see anything at all through the driving sleet and storm, it is a weary waste of roaring waters and crumbling, grating, crashing icebergs.

DIXON'S SCALPEL is devoted to the promotion and preservation of human health, and is edited by a man of acknowledged scientific abilities; it is a publication which, if distributed to every household in America, and even half followed out in its health teachings, would do more to happify our people (his cod-liver oil vagary excepted) than all the speeches of a Congressional session. We regret to learn that it has been published at a loss of two thousand dollars, and that, too when it is issued at a cost of *one dollar a year*, a sum so trifling as to place it within the ability of almost every family. Its teachings about health are too useful and true to command a remunerative subscription-list; because these are qualities which the present age does not seem inclined to patronize.

WHITEWASH adds so greatly to the picturesque in the cottage and the farm-house, and is such an absorbent of impure odors, that it should be freely used, at least in the spring. Take half a bushel of fresh-burned white lime, and slake it either with hot or cold water, in a tub or barrel. When thoroughly slaked, dissolve in the water required to thin the lime, two quarts of common salt, stir it thoroughly, add one quart of sweet milk, and it is ready for use, to put on with a brush, frequently stirring it up. Glues and gums cause it to scale off in hot weather.

TOO numerous to mention are the little conveniences of having a little flour PASTE always at hand, as those made of any of the gums impart a glaze to printed matter, and make it rather difficult to read. Dissolve a table-spoonful of alum in a quart of warm water, and when cold, stir in as much flour as will give it the consistency of thick cream, being particular to beat up all the lumps, then stir in as much powdered rosin as will stand on a dime, then throw in half a dozen cloves, merely to give a pleasant odor. Next, have a vessel on the fire which has a teacupful or more of boiling water, pour the flour mixture on the *boiling* water, stir it well all the time; in a very few minutes it will be of the consistence of mush; pour it out in an earthen or china vessel; let it cool; lay a cover on it, and put it in a cool place. It will keep for months. When needed for use, take out a portion and soften it with warm water. We keep ours covered an inch or two in water to prevent the surface from drying up. Paste, handled in this way, will last for twelve months.

OUR EXCHANGES.—Some of our most valued exchanges often fail to reach us for months together. We have received but one number of *Dixon's Scalpel.* The ever excellent *Arthur's Home Magazine* comes now and then, when we write for it. *The Home Journal*, so tastefully plain and beautiful mechanically—to say nothing of the chaste and entertaining variety of its reading matter—failed to reach us at all, in spite of constant complaints; and now we only get it by having it brought to our door. On several accounts we missed *Godey's Lady's Book*, most of all, for that beautiful trait, causing it to stand out shiningly, *alto relievo*, among the multitude of its contemporaries, in that it admits no profanities, and never, in more than a quarter of a century, has insulted any man's religious sentiment.

*Littell's Living Age*, with its large amount of valuable weekly reading, comes no longer. That quintessence of Calvinism, the *Princeton Review*, we have not seen for a year. The *Presbyterian Magagine*, monthly, of Philadelphia, reaches us about once semi-occasionally. We have always gone for our own papers, and mail the JOURNAL OF HEALTH to our exchanges ourselves. We cannot say where the fault of non-reception lies.

*Graham's Magazine* never fails to come up to time. The June number closes the *fifty-second* volume—a sign of stability and pecuniary thrift vouchsafed to very few publication enterprises of the present century.

If any crusty, grumpy, crotchety bachelor reader wants to be convinced of the folly of his ways, let him at once purchase the June number of *Arthur's Home Magazine*, and *Godey's Lady's Book*. The engravings of "The Happy Family," and "The First Step," are sweetly beautiful.

*The Eclectic Medical Journal* of Cincinnati, for May, contains a Valedictory Address to the Medical Graduates of Harvard University, by OLIVER WENDEL HOLMES—a name which needs no prefix or affix to give it designation—a name in medicine what that of HENRY CLAY was in national politics. It is one of the most manly and high-minded addresses of the kind we have ever read. It would make every doctor a gentleman.

Messrs. RUDD & CARLTON of New York, send "*A Woman's Thoughts about Women*," for one dollar. It promises much that women have thought, but which has never come to the surface in word or writing. It to old and young, because it is wisely practical, closing thus:

> "Only the memory of the just
> Smells sweet, and blossoms in the dust."

*Researches on Primary Pathology*, by M. L. KNAPP., M. D., first vol., 336 pages 8vo., treats of Cholera, Cholera Infantum, and Nursing Sore Mouth. The second vol. will treat of Scorbutic tendencies, aiming to prove the essential unity of disease. The author is a medical scholar, and has exhibited considerable research—bringing all his ability and knowledge to bear on one of the most vigorously disputed points in medicine, and one which has attracted a large amount of professional investigation.

*London Lancet* for April, $5 a year, 222 Broadway, abounds with items of medical news and progress. *Braithwaite's Retrospect* for the latter half of 1857, by the same house, is a volume of great interest. That ancient quarterly, *The Medico-Chirurgical Review*, republished with great promptness by the Messrs. WOOD, of this city, is filled with matter of a sterling character. $3 a year, in advance.

FLORA; OR, THE GIPSY'S FROLIC: *A Pastoral Opera, in Three Parts, Drama and Music*, composed and dedicated to the Century Club, by a fellow-member. To be rich by inheritance from early youth; to command the advantages of high social position, to possess and cultivate, with the ardor of an amateur, the refinements of Music, Poetry, and Painting, and all the while to hold one's-self aloof from the follies, and fripperies, and useless extravagances of the times—enjoying in the bosom of a loving family, wife, children, and friends, with the uninterrupted health of all for many successive years—the happiness not broken in upon by death, pecuniary reverse, or family misfortune—this is a lot not vouchsafed to more than one in a million. Dr. WARD owes it all to the rare wisdom of making himself satisfied with a moderate fortune of a few hundred thousand, and to the moral courage of educating himself and those around him to live according to the dictates of a rational physiology. In perfecting this genial and exhilarating opera, the generous and hospitable author and composer has been most lavish in his expenditures, in order to afford the highest satisfaction to some select friends at its first performance; and if he would follow the lead of his Knickerbocker wife, in her large and thoughtful care of the poor, we suggest, with *The Musical World*, that one or more performances be subsequently arranged as a medium of a princely offering of the richer public, to the children of poverty and misfortune—thus bringing the pastimes and pleasures of the rich under contribution to the lap of the poor, and making of high and low together one brotherhood.

A gentleman writing from Pennsylvania says: "I have never seen but two numbers of your JOURNAL, and I must say that I have never seen anything to compare with them."

*Challen's Monthly*, Philadelphia, has a series of most graphic and interesting papers on *European Life, Legend, and Landscape.* "*Grow Beautiful*," is another article which we read with considerable interest! Where did it come from? To grow old, and kindly, and good, altogether, is worth being known, it is the fit ending of a good life.

EMIGRATION.—We have long been impressed with the belief of the healthfulness of the climate of Minnesota; the long, dry, and steadily cold winters in localities free from much wind, must contribute to the restoration of persons laboring under consumptive tendencies. Travellers should make direct to *Superior City*, at the south-western extremity of Lake Superior—a locality which is destined to be the Chicago of the great Northwest. Persons desiring investments would do well to advise with some leading citizen of standing. JAMES M. ARNOLD, Esq., a gentleman of the old school, of education, and high social position, may be most safely consulted.

*The Printer*, monthly, $1 a year, 16 pp. quarto, No. 1 Spruce street, New York, is got up with great good taste, and promises to be useful to publisher and patron.

